# A Role for the Transcription Factor Nk2 Homeobox 1 in Schizophrenia: Convergent Evidence from Animal and Human Studies

**DOI:** 10.3389/fnbeh.2016.00059

**Published:** 2016-03-30

**Authors:** Eva A. Malt, Katalin Juhasz, Ulrik F. Malt, Thomas Naumann

**Affiliations:** ^1^Department of Adult Habilitation, Akershus University Hospital Lørenskog, Norway; ^2^Institute of Clinical Medicine, Ahus Campus University of Oslo Oslo, Norway; ^3^Institute of Clinical Medicine, University of Oslo Oslo, Norway; ^4^Department of Research and Education, Institution of Oslo University Hospital Oslo, Norway; ^5^Centre of Anatomy, Institute of Cell Biology and Neurobiology, Charite Universitätsmedizin Berlin Berlin, Germany

**Keywords:** brain development, GABA, NKX2-1, Schizophrenia, thyroid hormones, calcium, immune system, brain

## Abstract

Schizophrenia is a highly heritable disorder with diverse mental and somatic symptoms. The molecular mechanisms leading from genes to disease pathology in schizophrenia remain largely unknown. Genome-wide association studies (GWASs) have shown that common single-nucleotide polymorphisms associated with specific diseases are enriched in the recognition sequences of transcription factors that regulate physiological processes relevant to the disease. We have used a “bottom-up” approach and tracked a developmental trajectory from embryology to physiological processes and behavior and recognized that the transcription factor NK2 homeobox 1 (NKX2-1) possesses properties of particular interest for schizophrenia. NKX2-1 is selectively expressed from prenatal development to adulthood in the brain, thyroid gland, parathyroid gland, lungs, skin, and enteric ganglia, and has key functions at the interface of the brain, the endocrine-, and the immune system. In the developing brain, NKX2-1-expressing progenitor cells differentiate into distinct subclasses of forebrain GABAergic and cholinergic neurons, astrocytes, and oligodendrocytes. The transcription factor is highly expressed in mature limbic circuits related to context-dependent goal-directed patterns of behavior, social interaction and reproduction, fear responses, responses to light, and other homeostatic processes. It is essential for development and mature function of the thyroid gland and the respiratory system, and is involved in calcium metabolism and immune responses. NKX2-1 interacts with a number of genes identified as susceptibility genes for schizophrenia. We suggest that NKX2-1 may lie at the core of several dose dependent pathways that are dysregulated in schizophrenia. We correlate the symptoms seen in schizophrenia with the temporal and spatial activities of NKX2-1 in order to highlight promising future research areas.

## Introduction

Schizophrenia is a complex disorder that affects approximately 1% of the world's population over the age of 18. Although its heritability is estimated to be as high as 0.7–0.9 (Mulle, 2012), environmental and/or stochastic influences can still influence the pathogenesis of this disorder. The two-hit hypothesis, which has gained considerable support, suggests that an underlying genetic susceptibility, combined with a distinct developmental insult, i.e., an infectious or inflammatory episode, can prime an individual for a later event that ultimately leads to the onset of the full clinical syndrome (Bayer and Altman, 2009; Feigenson et al., 2014).

One of the fundamental goals in understanding schizophrenia is to link the observable symptoms to the underlying unobservable pathophysiology. The hallmark symptom is psychosis, with positive, negative, and cognitive manifestations (Andreasen et al., 1990). However, the majority of patients with schizophrenia receive neuroleptic treatment at an early stage of their illness. Accordingly, the fully evolved phenotype, as seen in untreated patients prior to the neuroleptic era, is seldom witnessed today. While being a welcome development for the patient, this can hamper our understanding of the key pathophysiological processes involved in schizophrenia. In this respect, a review of the remarkably detailed phenomenological studies of schizophrenic patients, conducted prior to the introduction of neuroleptics, is of particular value.

The most detailed observations of the evolving symptomatology in schizophrenia were made almost 100 years ago (Kraepelin, 1919). Kraepelin observed a number of non-psychiatric symptoms in his patients, indicating that several organ systems and physiological processes were affected.

Neurologic symptoms with spasmodic phenomena, reminiscent of choreic patients, were one of the observed dysfunctions. Later studies confirmed that children at high risk of developing schizophrenia often display neurological symptoms, including choreatic movements (Walker et al., 1994; McNeil et al., 2003). The presence and characteristics of these involuntary movements for patients with untreated psychosis, which disappear on treatment, indicate that these symptoms are an intrinsic feature of the disease process. Further, these involuntary movements imply a fundamental dysfunction in the cortical-basal ganglia-cortical circuitry (Whitty et al., 2009).

Frequent thyroid dysfunction, including an enlarged gland, rapid changes in size during progression of the illness, occasional exophthalmia, tremor, and myxoedema, have also been described (Kraepelin, 1919). A high incidence of thyroid pathology has been a consistent feature in reports of schizophrenia, with the available evidence supporting a role for thyroid hormone deregulation in the illness. The implication of thyroid hormone homeostasis in the fine-tuning of crucial brain networks would appear to warrant further research (Santos et al., 2012; Jose et al., 2015; Labad et al., 2016).

Furthermore, patients with schizophrenia present with somewhat accelerated and an excessively deep respiration, with several anomalies, especially for expiration (Kraepelin, 1919). Subsequently, it has been confirmed that patients with schizophrenia have an increased risk of lung disease, with a smoking-independent increase in lung disease-related mortality (Carney et al., 2006; Copeland et al., 2007).

Other symptoms and physiologic abnormalities initially described by Kraepelin, and subsequently confirmed by others, includes the following; impaired immunity (Anders and Kinney, 2015; Müller et al., 2015), enteric dysfunction (Peupelmann et al., 2009), altered calcium homeostasis (Bojarski et al., 2010), metabolic irregularities (Mitchell et al., 2013), skin abnormalities (Kamolz et al., 2003; Smesny et al., 2007); altered sleep patterns (Bromundt et al., 2011), an abnormal pupillary light reflex (Bar et al., 2008), altered thermoregulation (Chong and Castle, 2004), and menstrual patterns (Malik et al., 2011). Taken together, these observations indicate that, in addition to being a neurodevelopmental disorder, schizophrenia affects multiple physiologic processes that may lead to thyroid-, parathyroid- gastrointestinal and immune system dysfunction, respiratory symptoms, and skin abnormalities.

Although genetic findings consistently point to a role for genes involved in transcription/gene expression, neural development, neurotransmission, and immune function in schizophrenia, the vast majority of heritability still remains unexplained. However, an interesting insight has come from analyses of genome-wide association studies (GWASs) for complex disorders. Common single-nucleotide polymorphisms appear to be systematically enriched in transcription factor binding sites, particularly those active during fetal development (Maurano et al., 2012). Interestingly, the transcription factors whose activities may be altered, govern those physiologic processes relevant to the disease or trait under study.

In aiming to link the observable symptoms to the underlying unobservable pathophysiology of schizophrenia, we believe that a “bottom-up” approach where we track a developmental trajectory from embryology, to physiologic process and behavior, may be of value. In this fashion, we have come to recognize that the highly conserved transcription factor NK2 homeobox 1 (NKX2-1, encoded by *NKX2-1*; GENE ID 7080; chromosome 14q13), also called thyroid transcription factor 1, or thyroid-specific enhancer binding protein, possesses properties of particular interest for schizophrenia. Contrary to many transcription factors that are ubiquitously expressed, NKX2-1's expression is restricted to specific parts of the brain, the thyroid- and parathyroid glands, the skin, lungs, and enteric ganglia (Bingle, 1997; Suzuki et al., 1998a; Garcia-Barcelo et al., 2005; Boggaram, 2009; Germain et al., 2013; Fernandez et al., 2015). NKX2-1 governs multiple physiologic processes relevant to those somatic symptoms commonly seen in schizophrenia (as described). NKX2-1 also plays a central role in neurodevelopment and is essential for the formation and function of subgroups of neurons, glia, and functional neural networks that are affected in schizophrenia. This transcription factor also interacts with several susceptibility genes for schizophrenia, and is involved in gene-environment interactions with neurodevelopmental implications.

Further indications of the possible role of NKX2-1 in the pathophysiology of schizophrenia came from studies of families affected by inactivating mutations in NKX2-1. These result in a syndrome called brain-lung-thyroid disease, or benign hereditary chorea, which presents with impaired coordination, delayed speech development, neonatal pulmonary distress, and congenital hypothyroidism (OMIM#118700) (Krude et al., 2002; Glik et al., 2008; Yamada et al., 2009; Vloet et al., 2010; Monti et al., 2015; Peall and Kurian, 2015). Schizophrenia, psychosis, and behavioral disturbances, have all been reported in several of the reported pedigrees, suggesting that the association is not merely coincidental.

The scientific literature gives no indication that a large proportion of people with schizophrenia could carry a mutation or copy number variation in the NKX2-1 gene. However, the influence of a transcription factor on disease processes may not necessarily be directly linked to a mutation in the transcription factor itself. Dysregulated pathway activity, as a result of genetic or epigenetic alterations in downstream target genes, upstream regulators, transcriptional co-activators, or suppressors, may lead to genetic and phenotypic heterogeneity that ultimately manifests as a disorder. Accordingly, NKX2-1 may lie at the core of several dose dependent pathways that are dysregulated in schizophrenia. To further explore the possible role of NKX2-1 in schizophrenia, we correlated the symptoms seen in schizophrenia with the temporal and spatial activities of NKX2-1, in order to highlight promising future research areas.

## Neurodevelopment and brain function

**Figure 1:**
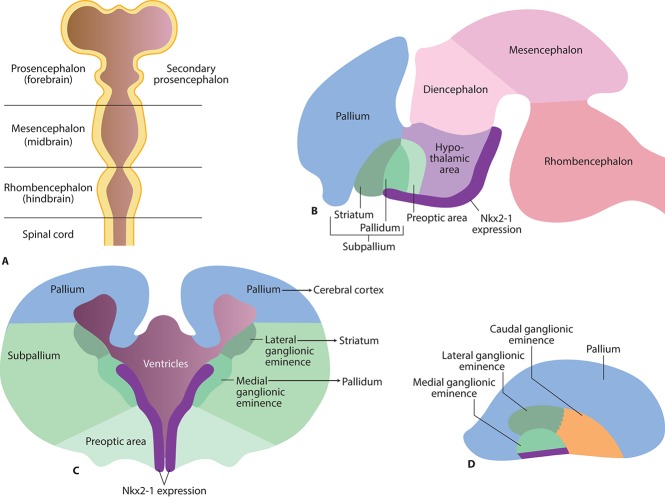
**Early brain development and Nkx2-1 expression in the early embryonic brain. (A)** Brain vesicles and divisions in the mammalian brain corresponding to gestational day 33 in humans. **(B)** Nkx2-1 is expressed as a longitudinal band in the ventral secondary prosencephalon from the rostral pallidum to the caudal hypothalamic area (rodent brain E15, corresponding to gestational day 49 in humans). **(C)** Future basal ganglia develop from subcortical parts of the telencephalon (“subpallium”). The striatum develops from the lateral ganglionic eminence, and pallidum from the medial ganglionic eminence. **(D)** Side view illustrating the medial, lateral, and caudal ganglionic eminences.

The neurodevelopmental hypothesis of schizophrenia proposes that pathological neurodevelopmental processes begin as early as the first and second trimester, and result in neuronal circuits that are primed to generate psychotic symptoms during adolescence or in young adults. In line with this, Nkx2-1 expression in the mouse brain commences at embryonic day 9 (comparable to the third gestational week in humans), when Nkx2-1 becomes detectable in a longitudinal band in the ventral forebrain that encompasses the medial ganglionic eminence (MGE), the preoptic area, and the ventral hypothalamus (Rakic and Zecevic, 2003a; Xu et al., 2008; Pauly et al., 2013) (Figure 1). Nkx2-1 participates in key early developmental events such as brain parcellation and cellular-fate specification in the embryonic forebrain (Sussel et al., 1999; Marin et al., 2000; Flames et al., 2007; Garcia-Lopez et al., 2008). Fate-mapping studies have shown that the progeny of Nkx2-1 expressing progenitor cells give rise to subclasses of neurons, astrocytes, and oligodendrocytes (Marshall and Goldman, 2002; Chojnacki and Weiss, 2004; Xu et al., 2008) while neurons derived from Nkx2-1-expressing progenitors are incorporated into specific neural circuits and networks, astrocytes and oligodendrocytes appear to be more widely distributed, and are found throughout the forebrain by the time of birth (Marshall and Goldman, 2002; Chojnacki and Weiss, 2004; Torigoe et al., 2015). Ultimately, in mice, the majority of Nkx2-1-expressing oligodendrocytes will be replaced by other populations of oligodendrocytes in the adult brain (Kessaris et al., 2006).

### Cortical gabaergic inhibition

Deficits in cortical GABA neurotransmission are among the most consistent findings in schizophrenia research (Inan et al., 2013). It has been suggested that this deficit results from dysfunctional GABAergic interneurons that share a common developmental origin, and possibly the same aberrant precursor pool (Fung et al., 2010). We would suggest that this population represents the progeny of NKX2-1-expressing progenitors, derived from the MGE, that then develop into specific subgroups of cortical GABAergic interneurons.

The GABAergic fate of Nkx2-1 expressing progenitor cells is determined by downstream activation of the transcription factor Lim homeobox 6 (Lhx6) (Du et al., 2008; Flandin et al., 2011). The continuous expression of Lhx6 in post-mitotic cortical interneurons allows us to trace their ancestry, even if Nkx2-1 expression is down regulated. Interestingly, a deficit in cortical *LHX6* mRNA expression has been reported in schizophrenic patients (Volk et al., 2012, 2014). In line with our suggestions, the authors propose that their finding implies a dysfunction in a GABAergic interneuron pool in schizophrenia.

Depending on temporal and spatial factors, Nkx2-1 derived, Lhx6 expressing GABAergic neuronal progenitors in the MGE may develop into subclasses of cortical GABAergic interneurons containing reelin, parvalbumin (PV+), somatostatin (SST+), calbindin (CB+), neuropeptide Y (NPY+), and nitric oxide synthase (NOS+) (Chojnacki and Weiss, 2004; Xu et al., 2004; Fogarty et al., 2007; Butt et al., 2008).

In rodents, early Nkx2-1-expressing progenitors in the MGE develop into a subpopulation of GABAergic reelin-containing Cajal-Retzius cells that will populate the (future) cortical layer I (Lavdas et al., 1999). Whether these will ultimately become reelin secreting cells is, as yet, unknown. Indirect evidence for dysfunctional reelin-containing cells in schizophrenia comes from studies of reeler mice, which are haploinsufficient for reelin, and share several biochemical and behavioral similarities with schizophrenic patients (Nullmeier et al., 2011). Among other abnormalities, reeler mice display impaired cortical lamination (Dekimoto et al., 2010). In line with this observation, patients with schizophrenia harbor an aberrant distribution of Cajal-Retzius cells and display a reduced expression of reelin in cortical layer I (Kalus et al., 1997; Ruzicka et al., 2007).

Some of the Nkx2-1 derived, reelin-secreting interneurons, also accumulate as interstitial cells in the medial regions of the subcortical white matter, at the midline in the corpus callosum, and in the hippocampus (DeDiego et al., 1994; Lavdas et al., 1999). In patients with schizophrenia, an aberrant distribution of interstitial cells and reduced reelin mRNA levels have been detected in the prefrontal cortex and in the hippocampal formation (Akbarian et al., 1996; Kirkpatrick et al., 2003; Eastwood and Harrison, 2006).

Although these findings may suggest that NKX2-1-derived Cajal-Retzius and interstitial cells are dysfunctional in patients with schizophrenia, it is important to bear in mind that these cell populations are heterogeneous, and have diverse molecular origins (Rakic and Zecevic, 2003b).

In mice, Nkx2-1-expressing precursors in the ventral MGE develop into PV+ interneurons that populate the cerebral cortex and the striatum. In the cerebral cortex, PV+ interneurons occur as basket and chandelier cells (Chojnacki and Weiss, 2004; Xu et al., 2004); approximately 90 and 68% of PV+ interneurons in the deep and superficial cortical layers, respectively, were co-labeled with reporter genes for Nkx2-1 (Xu et al., 2008).

Human and animal studies suggest that compromised cortical PV+ interneuron function is a major hallmark of schizophrenia (Reynolds et al., 2004; Behrens and Sejnowski, 2009; Curley et al., 2011; Nullmeier et al., 2011; Nakazawa et al., 2012; Volk et al., 2012; Jiang et al., 2013; Glausier et al., 2014; Yanagi et al., 2014; Brisch et al., 2015; Fujihara et al., 2015; Gonzalez-Burgos et al., 2015). Although most studies report functional impairment in cortical PV+ interneurons, some studies have also detected structural alterations (Konradi et al., 2011; Wang A. Y. et al., 2011).

In mice, most cortical SST+ interneurons develop from Nkx2-1-expressing progenitors in the MGE (Xu et al., 2004; Fogarty et al., 2007; Tricoire et al., 2011; Cai et al., 2013). In the neocortex, SST+ interneurons occur mainly as small basket cells in layers IV and V, and Martinotti cells in layers II-III and V-VI (Viollet et al., 2008). SST+ interneurons constitute 30–50% of all interneurons in the hippocampus, including bistratified, axo-axonic, and oriens-lacunosum-moleculare cells (Lawrence, 2008).

The cortical SST+ interneurons have been investigated less extensively than PV+ interneurons, but evidence is accumulating to show that their functions are also compromised in patients with schizophrenia (Hashimoto et al., 2008; Lewis et al., 2008; Morris et al., 2008; Fung et al., 2010; Beneyto et al., 2012). For example, the number of SST+ interneurons, as well as levels of *SST* mRNA, are both reduced in the hippocampus of schizophrenic patients (Konradi et al., 2011).

In mice, Nkx2-1-expressing progenitors in the preoptic area give rise to cortical CB+ interneurons (Fogarty et al., 2007). In the neocortex, most of this interneuron class occurs in the upper layers, often at the boundary between layers I and II (Gelman et al., 2009). Several studies have reported a reduction in the density of CB+ interneurons in the upper cortical levels in patients with schizophrenia (Beasley et al., 2002; Cotter et al., 2002; Reynolds et al., 2004; Chance et al., 2005; Sakai et al., 2008). Contradicting this, a 40% increase in the density of CB+ interneurons expressing the NR2A subunit of the N-methyl-D-aspartate receptor was reported for layer II of the anterior cingulate cortex in schizophrenic patients (Woo et al., 2008).

In rodents, cortical calretinin-containing interneurons do not develop from Nkx2-1-expressing progenitors. In humans, the NKX2-1 derived cortical calretinin-containing interneurons would appear to be a relatively minor subpopulation (Yu and Zecevic, 2011). Thus, far, the majority of studies have failed to identify any abnormality in calretinin-containing interneurons in schizophrenia (Beasley et al., 2002; Tooney and Chahl, 2004). However, decreased expression of calretinin mRNA in the dorsolateral prefrontal cortex of schizophrenia patients has been detected in a single study (Fung et al., 2010).

### Integration of information in cortical-basal ganglia-thalamocortical neural circuits

The basal ganglia receive afferent inputs from different areas of the cortex and send projections back to the cortex via the thalamus. This neuronal loop serves as the basis for various functions, including motor functions, cognitive control, motivational, and emotional processing. Early observations of involuntary movements in schizophrenic patients led researchers to suggest that the cortical-basal ganglia-thalamocortical circuits might be, in some way, dysfunctional (Whitty et al., 2009). It is now widely acknowledged that this dysfunction is actually widespread and contributes substantially to the pathophysiology of schizophrenia (Siegel et al., 1993; Ellison-Wright et al., 2008; Yoon et al., 2013; Cordon et al., 2015; Duan et al., 2015).

The progeny of Nkx2-1 expressing progenitors are widely distributed in cortical-basal ganglia-thalamocortical circuits. During early development, Nkx2-1 is expressed in both the dorsal and ventral parts of the striatum and pallidum. However, persistent expression into adulthood, is most prominent in the dorsal striatum and globus pallidus (Marin et al., 2000; Nobrega-Pereira et al., 2010).

The majority of GABAergic and cholinergic interneurons in the dorsal striatum in rodents are derived from the Nkx2-expressing progenitors in the MGE and preoptic area (Marin et al., 2000; Fragkouli et al., 2009; Magno et al., 2011). GABAergic interneurons belong to different subclasses containing PV, SST, nitric oxide synthase, neuropeptide Y, or calretinin (Marin et al., 2000), and modulate the output of neurons in the striatum (the medium spiny neurons) in a complex and highly orchestrated manner.

In humans, a major proportion of the NKX2-1 expressing striatal GABAergic interneurons contain PV (Magno et al., 2011). These constitute the major component of a powerful feed-forward inhibition that focuses cortical excitation, and synchronizes the activity of medium spiny neurons in order to regulate striatal output (Tepper et al., 2008). Although we have limited knowledge about the role of striatal GABAergic interneurons in schizophrenia, striatal PV+ interneurons have been suggested to play an important role in the behavioral effects mediated by antipsychotic drugs (Wiltschko et al., 2010). Indirect indicators of the influence of striatal PV+ interneurons in schizophrenia have also come from studies of reeler mice (Marrone et al., 2006; Ammassari-Teule et al., 2009). These reported a reduced number of striatal PV+ interneurons in all striatal sub regions, together with behavioral deficits such as fear extinction and latent inhibition.

In mice, at least 80% of striatal cholinergic interneurons develop from Nkx2-1-expressing progenitors (Fragkouli et al., 2009; Magno et al., 2009). Similarly, in the human brain, striatal cholinergic neurons are NKX2-1-immunoreactive (Magno et al., 2011). Although little is known about this interneuron group in schizophrenia, Holt et al. (1999, 2005) found that the mean density of cholinergic interneurons in the ventral striatum was reduced to just over a quarter (26%) of that seen in controls.

The globus pallidus is a rather homogenous structure composed of a network of inhibitory GABA-containing projection neurons that comprise the final pathway of the cortical output apparatus. In mice, approximately 75% of neurons in the globus pallidus develop from Nkx2-1-expressing progenitors that maintain their expression of Nkx2-1 into adulthood (Flandin et al., 2010; Nobrega-Pereira et al., 2010; Abdi et al., 2015; Dodson et al., 2015). These neurons are PV+ and display a spontaneous high-frequency discharge. The Nkx2-1 expressing neurons project through the direct and indirect pathway to the thalamus and the subthalamic nucleus. Their dendrites often cross functional borders within the pallidum to receive and integrate inputs from all parts of the pallidal complex (Bolam et al., 2000). In contrast, neurons that do not express Nkx2-1 send their projections back to striatum (Abdi et al., 2015).

To our knowledge, the projection neurons of the globus pallidus have not been directly studied in patients with schizophrenia. However, studies in an animal model based on the immunoinflammatory hypothesis of schizophrenia suggest that the globus pallidus may play crucial roles in the behavioral deficits seen in schizophrenia (Sotoyama et al., 2011). These include abnormal sensorimotor gating, latent inhibition, social interaction, working memory, and behavioral sensitization to dopamine and methamphetamine. In particular, the globus pallidus neurons in the lateral area display an increased firing rate that was ameliorated by treatment with risperidone (Sotoyama et al., 2013). Morphological and functional alterations of the globus pallidus have also been observed in patients with schizophrenia. Volumetric analyses, diffusion tensor imaging, and positron emission tomography, have revealed microstructural and functional alterations (Velakoulis et al., 2002; Galeno et al., 2004; Spaniel et al., 2005; Spinks et al., 2005; Hashimoto et al., 2009). There is also a strong positive correlation between markers of inflammatory and endothelial activation, and the volume of the globus pallidus in patients with schizophrenia (Dieset et al., 2015).

#### A putative special role for PV+ neurons in data integration

**Figure 2:**
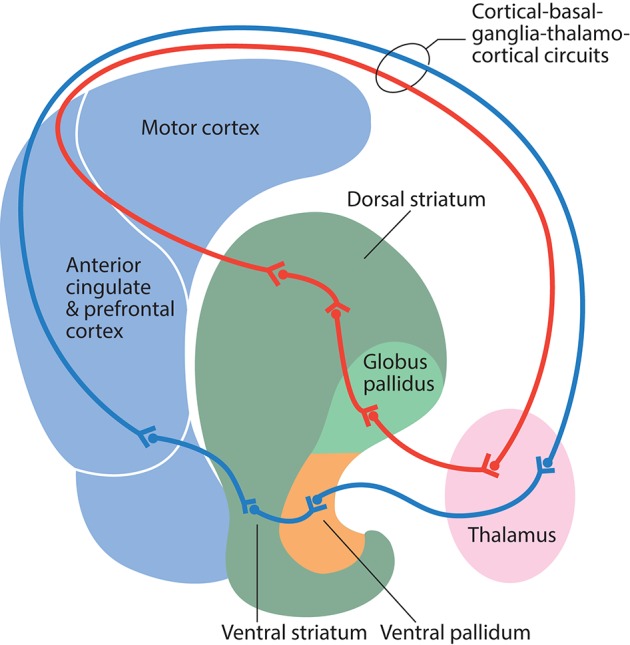
**Schematic illustration of information flow through the direct pathway in cortical-basal ganglia-thalamocortical circuits**. Information from the motor cortex passes to the dorsal striatum, globus pallidus, ventral lateral thalamus, and back to the motor cortex (red line). Information from the anterior cingulate and the prefrontal cortex passes to the ventral striatum, ventral pallidum, mediodorsal thalamus, and back to the anterior cingulate and prefrontal cortex (blue line).

The classic view of the organization of the basal ganglia is that functionally diverse information from the cerebral cortex is processed in the striatum and subsequent divisions of the basal ganglia by parallel and segregated circuits; these are organized in a dorsolateral to ventromedial gradient, from motor to limbic functions (Alexander et al., 1986) (Figure 2). However, context-dependent goal-directed patterns of behavior are increasingly recognized to be dependent on the temporal integration of limbic, associative, and motor information, rather than relying on spatial segregation (Bolam et al., 2000; Tisch et al., 2004).

We would suggest that the morphological organization and functional role of Nkx2-1-derived PV+ neurons in the rodent cortex, striatum, and pallidum, indicates that these neurons play a special role in data integration. Cortical PV+ interneurons receive their primary inputs from thalamocortical afferents, and integrate signals that converge at various parts of the cortical apparatus via their interneuron network. They mediate widespread perisomatic feed-forward inhibition to principal cortical neurons, and participate in the control of their output to the striatum (Mallet et al., 2005). Striatal PV+ interneurons receive convergent inputs from functionally different cortical territories and transmit integrated cortical information to the principal striatal medium spiny neurons (Ramanathan et al., 2002; Gage et al., 2010). PV+ projection neurons in the globus pallidus receive convergent inputs from all regions of the pallidal complex and integrate motor, associative, and limbic information (Bolam et al., 2000). The sum of their firing rate (which fluctuates) modulates the output of the basal ganglia (Elias et al., 2008) and thalamic activity. The thalamus completes the circuit by sending robust projections back to cortical PV+ interneurons and principal cells.

Rodent Nkx2-1 expression is developmentally regulated; correct spatial and temporal expression is required for the development and mature function of cortical-basal ganglia-thalamocortical circuits. Impaired Nkx2-1 expression in murine basal ganglia leads to substantial changes in coordinated movement (Magno et al., 2011). For normal mature function, rodent PV+ interneurons must down-regulate their expression of Nkx2-1 in the cortex, while achieving a mix of down-regulation and sustained expression in the allocortex. Conversely, continual expression of Nkx2-1 is required for the normal function of striatal PV+ interneurons and pallidal PV+ projection neurons in the basal ganglia (Xu et al., 2008; Magno et al., 2009).

Accordingly, any aberration in the developmental regulation of Nkx2-1 expression in PV+ neurons would alter information processing and integration in cortical-basal ganglia-thalamocortical circuits, with potentially far-reaching consequences for context-dependent goal-directed patterns of behavior. Such behavioral disturbances in patients with schizophrenia are well-known, having first been described in the pre-neuroleptic area (Kraepelin, 1919).

### Neural oscillations and neurogenesis

The functions most affected in schizophrenia require the large-scale integration of subsystems that use neural oscillations; these are thought to require a synchronization that enables flexible communication both within, and between, cortical areas. Impaired synchrony has emerged as a potentially fundamental pathophysiological mechanism in schizophrenia. Aberrant neural oscillations and their synchronization may account for enduring deficits in cognition, and some of the psychotic symptoms of the disorder (Uhlhaas and Singer, 2011). This idea is supported by accumulating evidence of abnormal theta and gamma activity in patients with schizophrenia (Schmiedt et al., 2005; Basar-Eroglu et al., 2008; Haenschel et al., 2009; Hamm et al., 2011; Gonzalez-Burgos et al., 2015).

Nkx2-1 expressing GABAergic and cholinergic neuronal clusters in the septal area, the diagonal band of Broca, the ventral pallidum, and the preoptic area play key roles in the generation of oscillatory activity (Morris and Henderson, 2000; Siapas et al., 2005; Hangya et al., 2009; Magno et al., 2009, 2011; Brockmann et al., 2011; McDonald et al., 2011; Griguoli and Cherubini, 2012). These clusters demonstrate topographically organized connections to various areas of the brain in order to influence arousal, sensory processing, emotion, motivation, learning, memory, and motor functions (Semba, 2000; Zaborszky et al., 2008).

The NKX2-1 expressing GABAergic basal forebrain neurons in humans and rodents have been identified as fast-spiking PV+ projection neurons (Freund, 1989; Magno et al., 2009). They exhibit burst firing at theta frequency and serve as pacemakers for the generation of cortical low-frequency theta oscillations (6–10 Hz; Morris and Henderson, 2000; Hangya et al., 2009; McDonald et al., 2011). In rats, hippocampal theta-burst activity, originating in the medial septum and ventral diagonal band, is known to drive oscillatory activity in the prefrontal cortex (Siapas et al., 2005; Brockmann et al., 2011), and is implicated in episodic and working memory, and the top-down control of cognitive functions (Uhlhaas et al., 2008). PV+ projection neurons in the basal forebrain can also regulate higher-frequency gamma-band oscillations (30–80 Hz), which are involved in higher cognitive functions such as feature binding, attention, and memory (Kim et al., 2015). The PV+ projection neurons in the medial septum and ventral diagonal band are also important for regulating hippocampal neurogenesis. For example, partial septohippocampal GABAergic denervation has been shown to reduce the survival of newly generated hippocampal neurons by approximately 40% in rats (Van der Borght et al., 2005). Collectively, data from human and animal research supports a link between schizophrenia and decreased hippocampal neurogenesis (Reif et al., 2006; DeCarolis and Eisch, 2010; Allen et al., 2015).

Activation of the Nkx2-1 expressing cholinergic projection neurons may change the direction of information flow within cortical circuits, and it has been suggested that the cholinergic projections support a common electrophysiological function in cortical target areas by increasing the amplitude and signal-to-noise ratio of sensory responses, while enhancing response selectivity (Castillo et al., 1999; Eggermann and Feldmeyer, 2009). This function has different effects on psychological processes depending on the neural network operations within the various cortical domains (Everitt and Robbins, 1997). The Nkx2-1 expressing cholinergic projection neurons in the rodent basal forebrain enhance GABAergic transmission in cortical interneurons, with a concomitant decrease in the firing of principal cells, thereby contributing to the promotion of low-frequency theta oscillations (Griguoli and Cherubini, 2012). In addition, cholinergic neurons recruit noncholinergic neurons that are required for hippocampal theta synchronization (Dannenberg et al., 2015).

The notion that basal forebrain cholinergic projection neurons may be dysfunctional in schizophrenia is supported by the finding that schizophrenic patients show less stable cortical signals with a lack of stimulus-related phase-synchronization of electromagnetic activity after stimulus presentation (Winterer et al., 2000). Patients tend to increase noise instead of building up a signal, thus generating a reduced signal-to-noise ratio during information processing. In support of this, a study of event-related blood-oxygen-dependent responses showed that noise variance strongly correlated with psychotic symptoms (Winterer et al., 2006).

The cholinergic neurons also play a role in spatial learning, which is impaired in mice after postnatal deletion of Nkx2-1-expressing cholinergic neurons in the basal forebrain (Magno et al., 2011). Spatial learning is known to be impaired among individuals with schizophrenia (Wilkins et al., 2013).

### Social processes and reproduction; a neuroendocrine network

Social recognition, affiliation, and attachment are all adversely affected in schizophrenia. Not surprisingly, the typical post pubertal onset of schizophrenia has stimulated interest in the potential contribution of neuroendocrine mechanisms and gonadal hormones (Walker and Bollini, 2002; Markham, 2012; Trotman et al., 2013).

**Figure 3:**
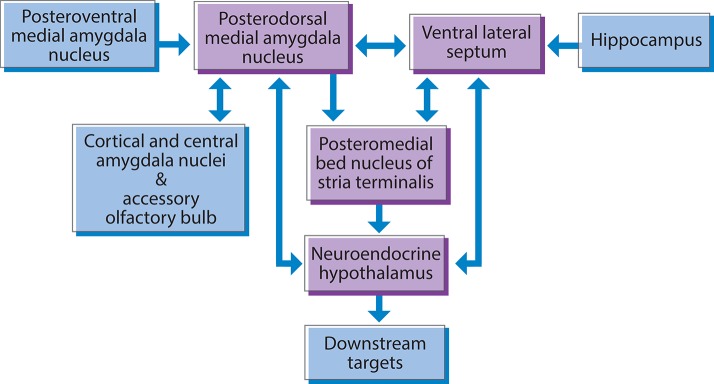
**Nkx2-1 is expressed in a subcortical neuroendocrine network involved in social behavior and reproduction in the rodent brain**. The posterodorsal medial amygdala, ventral lateral septum, and posteromedial bed nucleus of the stria terminalis, provide major inputs to the hypothalamic neuroendocrine effector system. Neuroendocrine hypothalamic nuclei provide feedback to the posterodorsal medial amygdala, and the ventral lateral septum.

Nkx2-1 displays a prominent lifelong expression in the highly interconnected neural neuroendocrine network that links sensory, hormonal, and homeostatic signals to social recognition and affiliation, reproduction, and parenting behaviors (Magno et al., 2009). This network includes subnuclei and cell groups in the amygdala, the lateral septum, the islands of Calleja, the hypothalamus, and the pituitary (Figure 3). As this network has been neatly delineated, it will be discussed in some detail.

**Figure 4:**
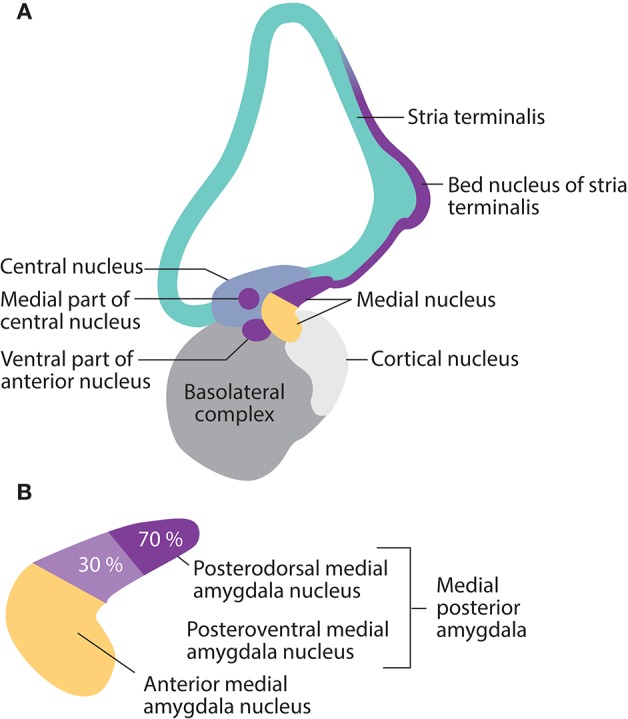
**Nkx2-1 expression in mature amygdala nuclei, and the stria terminalis in the rodent brain is restricted to subnuclei involved in social, reproductive, and fear-defense responses. (A)** Expression occurs in the posterodorsal and posteroventral medial nuclei, the ventral part of the anterior nucleus, the medial part of the central nucleus, the medial extended amygdala, and the posteromedial bed nucleus of the stria terminalis. **(B)** Details of the medial amygdala nucleus. Nkx2-1 is expressed by 70% of neurons in the posterodorsal medial nuclei (social-reproductive) and 30% of neurons in the posteroventral medial nuclei (fear-defense), suggesting different roles for NKX2-1 in the two sets of responses.

Cell groups that express Nkx2-1 are present in all parts of the amygdala complex during pre- and postnatal mouse development. In adults, Nkx2-1 expression is restricted to a small population of subnuclei. Its most pronounced expression is seen in subnuclei involved in the processing of olfactory and steroid hormone information into socio-sexual, neuroendocrine, and reproductive responses; these include the posterodorsal medial amygdala nucleus, the posteromedial bed nucleus of the stria terminalis, and the ventral anterior amygdala (Garcia-Lopez et al., 2008) (Figure 4). These subnuclei represent a pallidal-like output apparatus with projections to downstream targets in the neuroendocrine hypothalamus.

The lateral septum is a node responsible for integrating the cognitive and affective information that control behavioral responses to particular environmental stimuli (D'Anna and Gammie, 2009; Freiria-Oliveira et al., 2009; Lee and Gammie, 2009). Nkx2-1 is expressed in several proliferating domains in the rodent lateral septum during early development (Flames et al., 2007). In adult rodents, conspicuous expression of Nkx2-1 was detected in a group of large CB+ neurons in the ventral part of the lateral septum that projects to neuroendocrine hypothalamic subnuclei (Garcia-Lopez et al., 2008; Xu et al., 2008; Magno et al., 2009).

The islands of Calleja in the ventral striatum/pallidum constitute another olfactory and chemosensory neuroendocrine striato-pallidal system which impacts social affiliation, pair bonding, and reproduction. In rodents, some neuronal clusters persistently express Nkx2-1 (Fallon et al., 1983; Magno et al., 2009; Novejarque et al., 2011).

Neuroendocrine hypothalamic subnuclei involved in mating, fertility, reproduction, and the integration of reproduction with other homeostatic and environmental signals include the central part of the medial preoptic nucleus, the ventrolateral part of the ventromedial hypothalamic nucleus, the anteroventral periventricular nucleus, and the ventral premammillary nucleus (Lee et al., 2001; Magno et al., 2009; Matagne et al., 2012). In rodents, Nkx2-1 is highly expressed in the neuroendocrine hypothalamus. Nkx2-1 contributes to the control of sexual maturation in mice, with expression increasing abruptly at puberty in order to repress the transcription of genes that inhibit puberty (e.g., proenkephalin; Lee et al., 2001) and to activate genes that drive puberty (such as gonadotropin-releasing hormone) (Matagne et al., 2012). Nkx2-1 also participates in the maintenance of the female reproductive cycle, which necessitates changes in gonadotropin-releasing hormone (Ojeda et al., 2000, 2006; Mastronardi et al., 2006; Provenzano et al., 2010).

In the rat anterior pituitary, *Nkx2-1* mRNA is expressed in growth hormone/prolactin-containing neurons, where Nkx2-1 inhibits transcription of the gene encoding growth hormone, while activating transcription of the gene encoding prolactin. These observations suggest that Nkx2-1 plays a role in regulating the trans-differentiation of neurons containing these two hormones (Lee et al., 2007). In the posterior pituitary in rodents, Nkx2-1 expression is localized to specialized astrocytes, called pituicytes, which are involved in regulating the secretion of oxytocin and vasopressin into general circulation (Theodosis, 2002; Magno et al., 2009; Rosso and Mienville, 2009).

The neuroendocrine neural network is sexually dimorphic. It is enriched in gonadal steroid hormone receptors (Coolen and Wood, 1998; Spiteri et al., 2010; Griffin and Flanagan-Cato, 2011) as well as the neuropeptides vasopressin and oxytocin, which are important modulators of network activity (Gabor et al., 2012; Gur et al., 2014). The functional connectivity of this network has been characterized in sheep. During parturition, levels of oxytocin mRNA increase in the islands of Calleja, the medial amygdala, the posteromedial bed nucleus of the stria terminalis, the lateral septum, and the neuroendocrine hypothalamic nuclei (Broad et al., 1999).

Several lines of evidence indicate that the neuroendocrine network that links sensory, hormonal, and homeostatic signals to a broad range of social behaviors is adversely affected in schizophrenia. Developmental changes in both positive and negative schizophrenic symptoms increase exponentially as the individual passes through adolescence and approaches early adulthood. The age at which positive symptoms first appear differs between males and females, which may be due to later puberty and associated maturational processes in boys (Galdos and van Os, 1995). A negative correlation between menarche age and schizophrenia onset has also been reported by some investigators (Cohen et al., 1999), although these findings have yet to be replicated by others (Ruiz et al., 2000; Hochman and Lewine, 2004). However, higher negative-symptom scores and greater functional impairment have been associated with subjects who reported a later age at menarche (Hochman and Lewine, 2004).

Schizophrenia is also associated with gonadal and sexual dysfunction, as emphasized in a report describing sexual function in first-episode schizophrenia patients (Malik et al., 2011). Menstrual irregularities and amenorrhea associated with psychosis were described long before the introduction of neuroleptic drugs (Kohen and Wildgust, 2008). Elevated basal levels of prolactin and growth hormone, aberrations in neuroendocrine-stimulation tests, and abolished diurnal hormonal variation also occurs in neuroleptic-naive schizophrenic patients (Kahn et al., 1992; Warner et al., 1993; Rao et al., 1994; Muller-Spahn et al., 1998).

Investigations into the circulating levels of vasopressin and oxytocin in patients with schizophrenia have returned inconsistent results (Raskind et al., 1987; Legros et al., 1992). Plasma levels of oxytocin in patients with schizophrenia appear to be lower than in healthy individuals, particularly in patients exhibiting hyponatremic polydipsia and emotional deficit (Goldman et al., 2008). Lower endogenous oxytocin levels have consistently been associated with impaired social cognition, especially for lower-level processes such as facial-affect perception (Strauss et al., 2015).

Parenting behaviors, which are also mediated through the Nkx2-1-expressing neuroendocrine social-reproductive network in rodents, have been a major concern for female schizophrenia patients after giving birth. Mothers with schizophrenia ordinarily have difficulty with the practical aspects of caregiving and tend to interact less sensitively with their infants (Abel et al., 2005). Fathers with schizophrenia also experience difficulty with their parental role (Jungbauer et al., 2010).

The social-neuroendocrine network is also of interest with respect to pharmacological interventions in schizophrenia. Intranasal administration of oxytocin may improve social cognition and social skills in patients (Gibson et al., 2014; Mercedes Perez-Rodriguez et al., 2015; Rich and Caldwell, 2015). The islands of Calleja have received particular attention due to their dense innervation by dopaminergic projections and their high expression of dopamine D_3_ receptors. These receptors have been suggested to mediate the unique antipsychotic effects of clozapine and are currently a major target for drug-discovery programs related to schizophrenia (Guo et al., 1998; Davoodi et al., 2014). Abnormal hormone responses elicited by gonadotropin-releasing hormone have been detected in schizophrenic patients (Brambilla et al., 1976; Ferrier et al., 1983; Cantalamessa et al., 1985). Beneficial effects on positive and negative symptoms, as well as the exacerbation of schizoaffective psychosis, have also been described in case reports following treatment with gonadotropin-releasing hormone analogs (Soreni et al., 2004; Abu-Tair et al., 2007).

In summary, multiple lines of evidence indicate a disrupted social-neuroendocrine network in schizophrenia. Nkx2-1's prominent expression in this network indicates that this transcription factor may be an important modulator of network activity, and, as a result, could influence the physiological processes and social behaviors that are disturbed in patients with schizophrenia.

### Fear and defensive responses

Individuals who suffer from schizophrenia often exhibit marked and stable deficits in fear perception and recognition; it has been suggested that schizophrenic patients suffer from a general disconnect in the processing of danger signals between the central and autonomic systems (Williams et al., 2007). Although the most pronounced expression of Nkx2-1 is seen in the social neuroendocrine neural network, it is also highly expressed, in rodents, in the core of a subcortical neural network, which is involved in fear perception, defense, and escape responses. This network comprises the posteroventral medial amygdala, the anteromedial and posteromedial bed nucleus of the stria terminalis, some neuronal groups in the medial part of the ventral anterior, the medial central amygdala nucleus, and the hypothalamic dorsal premammillary nucleus (Choi et al., 2005; Garcia-Lopez et al., 2008; Magno et al., 2009; Carney et al., 2010) (Figure 4). Electrical stimulation of the hypothalamic part of this network elicits somatomotor and autonomic responses that resemble the behavior of animals facing natural threats. This fear network has been shown to be critical for the defensive responses (flight or freezing) elicited in animals in the presence of a predator (Canteras, 2002).

The investigation of fear-related behavior in patients with schizophrenia indicates that their fear system is hyper-activated. In a study of 1030 patients, 27% were found to exhibit “flight” behavior (Wang et al., 1997). In another study, of auditory hallucination, patients reported that they experienced these voices as powerful, dominating, and controlling; these provoked subordinate defensive responses, especially the fight/flight response (Gilbert et al., 2001). The initial “flight” behavior of patients in medical consultations, which is so frequently seen, has been suggested to be specific for schizophrenia, and is associated with the symptoms of the illness (Dimic et al., 2010).

In rats, the fear circuitry that we've just described mediates the freezing behavior that dominates fear responses to dominant males, and the risk assessment behaviors elicited by contextual fear of social defeat (Faturi et al., 2014). This is highly relevant for the social defeat hypothesis for schizophrenia, that posits that social defeat (i.e., the negative experience of being excluded from the majority group) is the common denominator of five major schizophrenia risk factors: urban upbringing, migration, childhood trauma, low intelligence, and drug abuse (Selten and Cantor-Graae, 2007; Selten et al., 2013).

The Nkx2-1 expressing, fear-defense related network, is so intimately connected, anatomically and functionally, with the social-neuroendocrine network, that activation of the fear network inhibits the social-neuroendocrine network. In a behavioral context this means that, upon the appearance of a threatening stimulus, a “gate control” mechanism ensures the rapid shut-down of the social-neuroendocrine network and associated behaviors in order to aid survival (Choi et al., 2005; Carney et al., 2010). Accordingly, a permanent hyper activation of the fear-defense network may have substantial implications for pro-social behaviors.

### Regulatory and homeostatic systems

#### Circadian rhythms, sleep and other effects of light

The influence of light on physiology and behavior (its so called masking behavior), extends to melatonin synthesis, daily activity, circadian rhythm, and sleep. Studies indicate that photic neurotransmission and photosensitive gene regulation are significant in schizophrenia and that the deregulation of masking behaviors are a common finding (Miller, 2013).

Melanopsin containing, adenylate cyclase-activating polypeptide producing intrinsically photosensitive retinal ganglion cells (ipRGCs), play a central role in transmitting photic information to non-visual target areas in the brain (Schmidt et al., 2011). Nkx2-1 is highly expressed in ipRGCs, where it is thought to regulate the transcription of adenylate cyclase-activating polypeptide, which is essential for the maintenance of photic sensitivity (Son et al., 2007; Kawaguchi et al., 2010).

The ipRGCs transmit daytime light information to the hypothalamic suprachiasmatic nucleus (SCN), and in doing so, contribute to circadian rhythms (Hattar et al., 2006). They also innervate several hypothalamic and pretectal areas involved in the control of masking behavior, regulation of the sleep-wake state, control of the pupillary light reflex, regulation of pineal melatonin levels, circadian oscillation of core body temperature, and neuroendocrine processes related to reproductive function (Hannibal, 2006; Hattar et al., 2006; Bailes and Lucas, 2010).

Nkx2-1 is also expressed in many of the down-stream targets that receive projections from ipRGCs, including the hypothalamic nuclei involved in the regulation of circadian rhythms: the dorsomedial hypothalamic nucleus and the SCN (Nakamura et al., 2001; Son et al., 2009). The dorsomedial hypothalamic nucleus is the key output nucleus of the brain's circadian system, as it is involved in circadian activation of a wide range of behavioral and endocrine functions (Chou et al., 2003). The SCN contains the circadian master clock. In the murine SCN, Nkx2-1 is expressed in neurons that express the protein, period-2 (Kim et al., 2002). In the rat SCN, period-1 and -2, additively stimulate the transcription of angiotensinogen, which integrates changes in the light-cycle with circadian variation in blood pressure and heart rate (Son et al., 2009). Essential elements of the master clock, such as the circadian locomotor output cycles kaput protein, and the aryl hydrocarbon receptor nuclear translocator-like protein, suppress Nkx2-1 expression (Kim et al., 2002). There is also evidence that Nkx2-1 is involved in generating a daily rhythm via its regulation of hypothalamic pituitary adenylate cyclase-activating polypeptide expression in rats (Kim et al., 2002). This process is required for the normal integration of the phase-advancing light signal (i.e., light induced changes to the circadian rhythm) by the SCN, and may be involved in synaptic plasticity in the hypothalamus (Gasperini et al., 2012).

The wide range of masking behaviors that are affected in schizophrenia indicate that the Nkx2-1 expressing neural network may be dysfunctional. Disturbances in circadian rhythm and the regulation of the sleep-wake state are generally observed and often appear as prodromal signs prior to the first and subsequently recurring psychotic episodes (Nishino et al., 2002; Karatsoreos, 2014; Manoach et al., 2014). Alteration in the synthesis and circadian rhythm of melatonin has also been reported (Monteleone et al., 1992, 1997; Rao et al., 1994). Compared to controls, patients with schizophrenia generally exhibit a deregulated body temperature, including a different baseline temperature, an abnormal daily temperature range, and an impaired ability to compensate for temperature stress (Chong and Castle, 2004; Shiloh et al., 2009). Significant alterations in pupillomotor control, with abnormal latencies and decreased light reaction, have also been reported (Bar et al., 2008). For example, a 10-fold higher stimulus intensity was required to induce a pupillary light reaction in patients with schizophrenia compared to controls (Rubin and Barry, 1976).

Although the major function of ipRGCs is to support non-image forming behaviors, their innervation pattern to the superior colliculus, the intergeniculate leaflet, and lateral geniculate nucleus, suggests that they relay inputs from the visual system to centers and circuits involved in the orientation of the head and eyes to sensory stimuli, image-forming vision, luminance, and spatial information (Ecker et al., 2010; Schmidt et al., 2011). This opens the possibility that abnormalities seen in early visual processing in schizophrenia might be influenced by Nkx2-1 expressing ipRGCs (Koychev et al., 2011; Khosravani and Goodarzi, 2013; Nunez et al., 2014; Lee et al., 2016).

#### Regulation of food intake

Long before the discovery of the neuroleptics, the altered consumption of food was noted in patients with schizophrenia, with substantial variations in food intake and large weight fluctuations over comparatively short periods of time (Kraepelin, 1919). Modern studies have also revealed that schizophrenia is often associated with eating disorders (Kouidrat et al., 2014). Anorexia nervosa affects between 1 and 4% of patients with schizophrenia. In addition, binge eating- and night eating disorders have an average prevalence of 5–20% in the schizophrenic population, which is approximately five fold that of the general population (Kouidrat et al., 2014). The mechanisms behind these altered eating behaviors are poorly understood.

One of the hypothalamic nuclei with a high continuous expression of Nkx2-1 is the arcuate nucleus, the master hypothalamic center for feeding control (Kim et al., 2002, 2006, 2011; Yee et al., 2009; Kaji and Nonogaki, 2013). In rodents, Nkx2-1 is selectively expressed in the two main centrally projecting cell populations involved in the regulation of feeding behavior; neurons containing the agouti-related protein and neuropeptide Y, and neurons containing peptide products of pro-opiomelanocortin and the cocaine and amphetamine-regulating transcript (Kim et al., 2006; Yee et al., 2009; Kaji and Nonogaki, 2013). Nkx2-1 stimulates transcription of agouti-related protein and inhibits transcription of the gene encoding pro-opiomelanocortin; up-regulation of Nkx2-1 has also been shown to increase appetite and eating (Kim et al., 2011).

Neuroleptic drugs are associated with varying degrees of weight gain, and direct or indirect effects on the hypothalamic neuronal circuits that control food intake and satiety represent plausible causal mechanisms for these side-effects (Kouidrat et al., 2014). Sub-chronic exposure to olanzapine, which leads to hyperphagia and weight gain in rats, is associated with the up-regulation of agouti-related protein/neuropeptide Y, and down-regulation of the pro-opiomelanocortin/cocaine and amphetamine-regulating transcript in the arcuate nucleus (Ferno et al., 2011; Weston-Green et al., 2012). Given that Nkx2-1 ordinarily regulates the balance between these two proteins, the possibility exists that olanzapine's side effects related to hyperphagia and weight gain may be mediated by up-regulated Nkx2-1. As yet, this possibility has not been explored.

#### Osmoregulation, water balance, and sympathetic outflow

Patients with schizophrenia often display alterations both in central and peripheral water balance. Multiple studies of the brains of patients with schizophrenia have uncovered abnormalities in their volume of cerebrospinal fluid. In a meta-analysis, ventricular cerebrospinal fluid volume was 20–30% higher in schizophrenic patients (Wright et al., 2000). A smaller increase of 7% was reported in a large Finnish cohort study which took into account both the ventricular and external volumes of cerebrospinal fluid (Tanskanen et al., 2009). In rats, Nkx2-1 has a regulatory role in the formation of cerebrospinal fluid. Nkx2-1 is expressed in the apical membrane of the ventricular choroid plexus, where it regulates expression of the water-channel protein aquaporin-1(Kim et al., 2007). This protein plays an essential role in the homeostasis of intracellular and extracellular water in the brain, and blockade of Nkx2-1 decreases cerebrospinal fluid formation by approximately 20% (Kim et al., 2007).

Deregulation of body-fluid homeostasis is also a well-known phenomenon in patients with schizophrenia. Primary polydipsia is the most common physiologic abnormality and may be present in more than 20% of chronic inpatients (de Leon et al., 1994). Increased sensitivity to osmotic stress and water intoxication due to polydipsia have often been described (Hundt et al., 2001; Bralet et al., 2007; Goldman et al., 2007; Satoh et al., 2007; Siegel, 2008; Goldman, 2009). In rats, Nkx2-1 influences water intake (Son et al., 2003). Nkx2-1 is expressed in the circumventricular organs; the organum vasculosum of the lamina terminalis, and the subfornical organ (Kim et al., 2008). Nkx2-1 activates transcription of the gene encoding angiotensinogen after water deprivation or dehydration, which influences drinking behavior.

Patients with schizophrenia regularly exhibit altered regulation of sympathetic outflow (Bar et al., 2007; Chang et al., 2009). Nkx2-1 is indirectly involved in autonomic regulation, as a major central target for angiotensinogen from the subfornical organ is the hypothalamic paraventricular nucleus, where it is converted to angiotensin II. Angiotensin II in the paraventricular nucleus increases the activity of the brain renin-angiotensin system and contributes to autonomic output and sympathoexcitation (Ferguson and Bains, 1997; Kang et al., 2009). Angiotensinogen from the subfornical organ also influences neuroendocrine secretion of adrenocorticotropic hormone, oxytocin, and vasopressin from the pituitary gland (Bartanusz and Jezova, 1994; Macova et al., 2009).

#### Integration of trophic and metabolic processes and the regulation of brain-body homeostasis

In mice, Nkx2-1 demonstrates a high, lifelong expression in tanycytes, although, to our knowledge, the role of Nkx2-1 in these cells has not yet been explored. Tanycytes are specialized astrocytes located in the periventricular area that bridges the lumen of the third ventricle with the blood vessels of the medial basal hypothalamus (Lee et al., 2001). Their strategic proximity to, and relationship with, fenestrated capillaries, the blood brain barrier, axonal nerve terminals, and hypothalamic nuclei that regulate appetite/energy expenditure, places them in a privileged position with which to integrate multiple inputs and regulate homeostasis (Goodman and Hajihosseini, 2015). Although much is still unknown about the specific roles of tanycytes, they are known to regulate the release of gonadotropin-releasing hormone and to participate in the regulation of the hypothalamo-pituitary-gonadal axis (Fekete and Lechan, 2014). They may also act as glucosensors and participate in the control of insulin secretion (Frayling et al., 2011). Tanycytes function as gatekeepers of thyroid-hormone level in the hypothalamus and regulate the hypothalamic-pituitary-thyroid axis during fasting and infection (Lechan and Fekete, 2007; Herwig et al., 2008; Fonseca et al., 2013; Fekete and Lechan, 2014). Currently, there are no reports about tanycytes in schizophrenia. However, their role in body-brain communication and homeostatic regulation should make them of interest in schizophrenia research.

## Thyroid hormone homeostasis

The available evidence supports thyroid hormone deregulation as a common feature in schizophrenia (Santos et al., 2012). In humans, NKX2-1 is necessary for organogenesis and the development of the thyroid gland (Kimura, 1996; Suzuki et al., 1998a; Damante et al., 2001). NKX2-1 makes major contributions to the maintenance of normal thyroid-hormone homeostasis by regulating expression of the thyrotropin-stimulating hormone receptor, thyroglobulin, thyroid peroxidase, the sodium/iodide symporter, and type II iodothyronine deiodinase (Berg et al., 1996; Endo et al., 1997; Suzuki et al., 1999; Damante et al., 2001; Gereben et al., 2001; Moeller et al., 2003).

### Thyroid hormones and the brain

Thyroid hormones exert profound neurodevelopmental effects; even modest disruption during critical periods of fetal development can influence mature brain function (Zoeller and Rovet, 2004; Oerbeck et al., 2007; Flamant et al., 2015). Triiodothyronine depletion results in the delayed expression of oligodendrocyte-specific markers, fewer oligodendrocyte cell bodies in the main white-matter tracts, delayed expression of genes encoding structural myelin proteins, fewer myelinated axons, and a lower myelin content (Valcana et al., 1975; Ibarrola and Rodriguez-Pena, 1997; Mohacsik et al., 2011). The maturation of GABAergic PV+ neurons depends heavily on thyroid hormones; deficiency during the early postnatal period in rats (corresponding to 5–6 months of gestation in humans) reduced PV immunoreactivity in GABAergic interneurons in the adult neocortex and hippocampus, leading to a compromised inhibitory function (Gilbert et al., 2007). Adult neural stem cell cycling and the maintenance of hippocampal pyramidal neuron populations depend on the modulation of specific cell-cycle regulators by thyroid hormones (Lemkine et al., 2005; Alva-Sanchez et al., 2009). Thyroid hormones also affect synaptic proteins (Yang et al., 2012) and glucose metabolism (Bauer et al., 2009), and protect the brain from oxidative stress by maintaining glutathione homeostasis (Dasgupta et al., 2007).

Brains from patients with schizophrenia display alterations that are compatible with the pre- and postnatal depletion of thyroid hormones. These alterations include oligodendrocyte abnormalities, defects in white matter tracts, and the dysfunction of PV+ GABAergic interneurons (Behrens and Sejnowski, 2009; Melicher et al., 2015). There are also findings suggestive of reduced neural stem-cell proliferation (Reif et al., 2006), presynaptic dysfunction (Castillo et al., 2010), and deficits in glucose metabolism and glutathione homeostasis (Katz et al., 1996; Gysin et al., 2007; Do et al., 2009; Yao and Keshavan, 2011).

### Thyroid autoimmune disease

Data from Danish registries show that the incidence of Graves' disease and autoimmune thyroiditis is increased in patients with schizophrenia and their parents, and that a history of any autoimmune disease is associated with a 45% increase in the risk of schizophrenia (Eaton et al., 2006; Benros et al., 2014). Autoimmune thyroid disease can be associated with other autoimmune endocrine failures or non-endocrine diseases (vitiligo, pernicious anemia, myasthenia gravis, autoimmune gastritis, celiac disease, hepatitis; Wemeau et al., 2013). Several of these disorders have also been associated with schizophrenia (Zoabi et al., 2012; Benros et al., 2014).

Autoimmune thyroid disease is provoked by a loss of self-tolerance to the autoantigens thyroid peroxidase thyroglobulin, and thyroid stimulating hormone receptor, which leads to a destructive immune infiltration of the gland. NKX2-1 promotes the expression of these thyroid autoantigens, and the up-regulation of NKX2-1 results in a concomitant increase in human leukocyte antigen (or major histocompatibility complex (MHC) class I expression, together with the thyroid autoantigens. This may constitute the pathologic state that leads to thyroid autoimmune disease (Huang et al., 2011). Bacterial lipopolysaccharide, which has been proposed to be another etiopathogenic agent in autoimmune disease, also up-regulates the expression of thyroid antigens by increasing Nkx2-1 expression in rats (Velez et al., 2006). It has recently been shown that the strong genetic association between variation in the MHC locus and schizophrenia arises, in part, from the many structurally diverse alleles of the complement component 4 (*C4*) gene (Sekar et al., 2016). Autoimmune thyroid disease can also be mediated by direct binding of complement C4 to thyroid peroxidase (Blanchin et al., 2003). Whether NKX2-1 is directly involved in this process has yet to be explored.

## Calcium homeostasis and bone cell metabolism

Research has demonstrated that impaired calcium homeostasis and signaling is common to many schizophrenia-related processes, including dysregulated dopamine and glutamate neurotransmission (Bergson et al., 2003; Martins-de-Souza et al., 2009a,b; Bojarski et al., 2010). Platelets from drug-free schizophrenia patients harbor significantly higher levels of cytosolic calcium than healthy controls (Ripova et al., 1997); data from a recent GWAS also supports the involvement of calcium-signaling in schizophrenia (Hertzberg et al., 2015).

Chief cells in the parathyroid gland coordinate with thyroid neuroendocrine C cells to maintain calcium and phosphorus levels in the blood, as well as bone-cell metabolism throughout the body. C cells secrete calcitonin, and in rats, Nkx2-1 activates transcription of calcitonin (Suzuki et al., 1998b, 2007). Calcitonin decreases serum calcium levels, increases calcium levels in cerebrospinal fluid, increases serum concentrations of 1,25-dihydroxyvitamin D, inhibits bone resorption, and regulates phosphorus metabolism (Carmen and Wyatt, 1977; Felsenfeld and Levine, 2015). In patients with schizophrenia, injections with synthetic salmon calcitonin have been shown to decrease agitation, and improve malignant catatonia (Carmen and Wyatt, 1977; Carman and Wyatt, 1979a, Carman and Wyatt, 1979b).

Nkx2-1 is also expressed in rat chief parathyroid cells (Suzuki et al., 1998a), with binding sites for Nkx2-1 present within genes encoding the calcium sensor receptor and calmodulin. Nkx2-1 may therefore act as an important calcium-sensing factor that responds to altered intracellular calcium levels in order to regulate the gene expression required to maintain calcium homeostasis (Suzuki et al., 1998b). Animals with abnormal calcium homeostasis, after removal of the parathyroid gland, exhibit changes in basal dopamine and noradrenaline levels in limbic structures, abnormalities in conditioned reflex activity, and the acquisition of adaptive behavioral strategies (Sashkov et al., 2007).

Regulation of bone formation and turnover is a main function of chief and parafollicular cells. Bone metabolism has been shown to be disturbed in patients with schizophrenia and decreased bone mineral density and osteoporosis are common in these patients. Schizophrenia has been found to be an independent determinant of a poor skeletal status in women after controlling for common risk factors such as osteoporosis, vitamin D status, and medication (Partti et al., 2010). From a young age, both men and women with schizophrenia present a lower bone mass than the general population (Renn et al., 2009).

## The respiratory system

Patients with schizophrenia have significantly lower lung-function values than control subjects after adjustment for smoking and other potential confounders (Partti et al., 2015). They experience a higher incidence of lung disease such as asthma, chronic obstructive pulmonary disease, and pneumonia (Carney et al., 2006; Filik et al., 2006; Copeland et al., 2007; Chen et al., 2009). Schizophrenic patients aged 10–39 years and 40–49 years are eight times and five times more likely, respectively, to die from respiratory causes (Partti et al., 2015).

NKX2-1 is highly expressed in the lungs, where it regulates the expression of several genes critical to lung development and mature function (Bohinski et al., 1994; Li et al., 2000, 2002; Naltner et al., 2000; Yuan et al., 2000; Berhane and Boggaram, 2001; Zhou et al., 2001; Reynolds et al., 2003; Zhu et al., 2004; Reynolds and Hoidal, 2005; Sparkman et al., 2006; Besnard et al., 2007; Spiteri et al., 2007; Minoo et al., 2008; Boggaram, 2009; Kolla et al., 2009; Tagne et al., 2012; Li C. et al., 2013; Zscheppang et al., 2013). In the adult mouse lung, Nkx2-1 is expressed predominantly in two special cell types, the alveolar type II epithelial cell, and Clara cells (Boggaram, 2009).

Alveolar type II cells are responsible for the production of surfactant; in rodents, rabbits and humans, Nkx2-1/NKX2-1 positively regulates the expression of surfactant proteins A, B, and C (Margana et al., 2000; Yi et al., 2002; Alcorn et al., 2004; Liu et al., 2008; Cao et al., 2010). Surfactant protein A interacts with a wide range of pathogens to suppress microbial growth, damage bacterial membranes, modulate macrophage phagocytosis, clear lipopolysaccharide, and regulate complement activation (Kishore et al., 2006). Surfactant proteins B and C are critical in lowering the surface tension at the air/water interface, and for airway host defense (Chroneos et al., 2010). Abnormal expression and activity of surfactant proteins have been suggested to underlie the pathogenesis of a variety of lung inflammatory diseases, including asthma and chronic obstructive pulmonary disease (Kishore et al., 2006; Chroneos et al., 2010). Consistent with this hypothesis, patients with heterozygous loss-of-function mutations in *NKX2-1* are predisposed to neonatal respiratory distress and pulmonary infections due to reduced expression of surfactant proteins (Krude et al., 2002).

Clara cells located in the bronchioles secrete surfactant protein B and Clara cell secretory protein (CCSP), both of which are transcriptional targets of NKX2-1 (Ray et al., 1996; Cassel et al., 2000; Margana et al., 2000). CCSP is a natural immuno-suppressant and anti-inflammatory secretory protein that exhibits several immunomodulatory features, including helper T-cell regulation (Dierynck et al., 1996; Hung et al., 2004; Johansson et al., 2007). Reduced plasma CCSP levels have been reported in patients with schizophrenia, and it has been suggested that inflammatory responses in schizophrenia may be causally related to lower serum CCSP levels, which may represent a biomarker for the disorder (Maes et al., 1996a,b; Lin et al., 1998).

## The enteric nervous system

Gastric dysmotility and enteric nervous-system dysfunction are common in schizophrenic patients (Peupelmann et al., 2009; Berger et al., 2010). In humans, NKX2-1 is expressed in mature enteric ganglia, where it regulates expression of the ret proto-oncogene by interacting with the transcription factors paired-like homeobox 2B, and sex-determining region Y-box 10 (Leon et al., 2009). Both of these are genes have been associated with susceptibility to schizophrenia (Ide et al., 2005; Maeno et al., 2007b; Glessner et al., 2010).

## The skin

Patients with schizophrenia exhibit skin abnormalities such as reduced wound healing and an attenuated response to inflammatory stimuli in the niacin skin-flushing test (Kamolz et al., 2003; Smesny et al., 2007). In rats, Nkx2-1 is expressed in keratinocytes and dermal fibroblasts (Suzuki et al., 1998a). While targets for Nkx2-1 in the skin have yet to be explored, genes related to thyroid hormones, inflammation, and calcium homeostasis, that are regulated by Nkx2-1 in other tissues, are also expressed in keratinocytes and dermal fibroblasts (Cianfarani et al., 2010; Yun et al., 2011). Skin keratinocytes and fibroblasts are also generally considered to be a useful model system for studying vulnerability factors and the pathophysiology of schizophrenia (Ramchand et al., 1995; Catts et al., 2006; Olsson et al., 2006; Gysin et al., 2009; Wang et al., 2010).

## Discussion

### NKX2-1 and candidate schizophrenia genes

A review of the publically available online database for genes associated with Schizophrenia (SchizophreniaGene) in December 2015, revealed in excess of 1000 possible candidate genes; this database was, until Dec. 2011, regularly updated (Allen et al., 2008). Some of these candidates were identified during the pre-GWAS period, with others based on GWAS studies. NKX2-1 is known to interact with a number of these genes (Thome et al., 1996; Perrone et al., 1999; Stober et al., 1999; Marin et al., 2000; Naltner et al., 2000; Ojeda et al., 2000; Stoykova et al., 2000; Kim et al., 2002; Leonard et al., 2002; Yi et al., 2002; Chojnacki and Weiss, 2004; Cui et al., 2005; Ide et al., 2005; Reynolds and Hoidal, 2005; Aghajani et al., 2006; Sanjuan et al., 2006; Takahashi et al., 2006; Benzel et al., 2007; Hashimoto et al., 2007; Son et al., 2007; Takao et al., 2007; Kahler et al., 2008; Zhou et al., 2008; Leon et al., 2009; Carney et al., 2010; Waddington et al., 2010; Goulburn et al., 2011; Zhang et al., 2011; Crisafulli et al., 2012; Matagne et al., 2012; Quednow et al., 2012; Li T. et al., 2013; Trotman et al., 2013; Zscheppang et al., 2013; Luo et al., 2014) (Table 1).

**Table 1:** **Genes identified to interact with NKX2-1/Nkx2-1 in human or rodent studies that have also been associated with susceptibility for development of schizophrenia or with the course of illness**.

**Gene name**	**Gene symbol**	**Species**	**Tissue**	**Process**	**Selected references**
Adenylate cyclase activating polypeptide 1	*Adacyp1^a^*	R	Retina, hypothalmus	Circadian rhythm, synaptic plasticity	Kim et al., 2002; Hashimoto et al., 2007; Son et al., 2007
Adenomatous polyposis coli	*Apc^b^*	M	Lung	Neuroendocrine cell differentiation	Cui et al., 2005; Li C. et al., 2013
Calreticulin	*CALR^b^*	H	Thyroid, lung	Immune system regulation	Perrone et al., 1999; Aghajani et al., 2006; Takahashi et al., 2006
Cholinergic receptor, nicotinic, alpha 7	*Chrna7^a^*	M	Lung	Lung development	Leonard et al., 2002; Reynolds and Hoidal, 2005; Luo et al., 2014
Clock circadian regulator	*Clock^b^*	R	Hypothalamus	Circadian rhythm	Kim et al., 2002; Takao et al., 2007; Waddington et al., 2010; Zhang et al., 2011; Matagne et al., 2012
CREB binding protein	*CREBBP^c^*	H	Lung	Lung development and function	Naltner et al., 2000; Yi et al., 2002; Crisafulli et al., 2012
Ciliary neurotrophic factor	*CTNF* ^c^	H	Forebrain	Astrocyte formation	Thome et al., 1996; Chojnacki and Weiss, 2004
Distal-less homeobox 1	*Dlx1^c^*	M	Forebrain	Ventral forebrain development	Marin et al., 2000; Kahler et al., 2008; Goulburn et al., 2011
V-erb-b2 avian erythroblastic leukemia viral oncogene homolog 2	*ErbB2^a^*	R	Hypothalamus	Central regulation of puberty	Ojeda et al., 2000; Benzel et al., 2007
V-erb-b4 avian erythroblastic leukemia viral oncogene homolog 4	*ErbB4^a^*	M	Forebrain, hypothalamus, lung	Interneuron migration and maturation, central regulation of puberty, lung cell development	Benzel et al., 2007; Zscheppang et al., 2013
Forkhead box P2	*FOXP2^b^*	M	Forebrain, lung	Emotional responses, surfactant production	Sanjuan et al., 2006; Zhou et al., 2008; Carney et al., 2010; Li T. et al., 2013
Paired box 6	*PAX6^c^*	H	Forebrain	Dorso-ventral brain patterning	Stober et al., 1999; Stoykova et al., 2000
Paired-like homeobox 2b	*PHOX2B^c^*	H	Forebrain, enteric ganglia	Oligodendrocyte development, enteric nervous system development, gut function	Ide et al., 2005; Leon et al., 2009
Proenkephalin	*Penk^a^*	R	Hypothalamus	Central regulation of puberty	Mikesell et al., 1996; Ojeda et al., 2000
Retinoic acid receptor, alpha	*Rara^c^*	M	Lung	Protein-protein interactions to enhance transcription	Yang et al., 2004; Rioux and Arnold, 2005
Ret proto-oncogene	*RET^c^*	H	Enteric ganglia	Enteric nervous system development, gut function	Leon et al., 2009; Glessner et al., 2010
SRY (sex determining region Y)-box 10	*SOX10^c^*	H	Brain, enteric ganglia	Oligodendrocyte development, gut function	Maeno et al., 2007a; Leon et al., 2009; Yuan et al., 2013
Sp1 transcription factor	*SP1^b^*	H	Lung	Lung development, host defense	Li et al., 2000; Pinacho et al., 2013
Sp3 transcription factor	*SP3^b^*	H	Lung	Lung development, host defense	Li et al., 2000; Pinacho et al., 2013
Sp8 transcription factor	*Sp8^c^*	M	Forebrain	Dorsoventral patterning, interneuron formation	Zembrzycki et al., 2007; Kondo et al., 2013
Transcription factor 4	*Tcf4^c^*	M	Forebrain	Formation of cortical GABAergic interneurons	Tebar et al., 2001; Lennertz et al., 2011; Wang H. et al., 2011; Quednow et al., 2012
Transforming growth factor, beta 1	*Tgfb1^b^*	M	Lung	Inflammation	Li et al., 2002; Frydecka et al., 2013
Tumor necrosis factor	*Tnf^b^*	M	Lung	Inflammation	Das et al., 2011; Paul-Samojedny et al., 2013

a *Downstream target;*

b *Upstream regulator;*

c *Other interaction*.

*M, Mouse; R, Rat; H, Human*.

In a recent publication, Farrell et al. (2015) presented genomic evidence for 25 candidate schizophrenia genes identified during the pre-GWAS period, including Disrupted in schizophrenia 1, Neuregulin 1, Nicotinic cholinergic receptor α7, and Receptor-erb-b4 avian erythroblastic leukemia viral oncogene homolog. Several of these genes, and their gene products, influence or are influenced by, Nkx2-1. Disrupted in schizophrenia 1 is necessary for the migration of Nkx2-1-derived interneurons to the cortex (Steinecke et al., 2014), and for mature function of the PV+ subgroup (Sauer et al., 2015). Disrupted in schizophrenia 1 is also involved in adult neurogenesis (Jun et al., 2012), a process in which Nkx2-1-expressing GABAergic PV+ projection neurons in the medial septum/ventral diagonal band play a key role (Van der Borght et al., 2005). Neuregulin 1, brain-derived neurotrophic factor, and dystrobrevin binding protein 1, are also of critical importance for the maturation and inhibitory function of Nkx2-1-derived PV+ neurons (Hashimoto et al., 2005; Grabert and Wahle, 2008; Fazzari et al., 2010; Carlson et al., 2011; Yin et al., 2013). Neuregulin 1 and nicotinic cholinergic receptor α7 are involved in the modulation of Nkx2-1-dependent hippocampal gamma oscillations (Fisahn et al., 2009; Lu and Henderson, 2010), as well as regulation of plasticity of the airway epithelium (Maouche et al., 2009). In the lungs of mice, signaling via neuregulin 1, and its receptor-erb-b4 avian erythroblastic leukemia viral oncogene homolog, activates Nkx2-1 expression (Zscheppang et al., 2013), while Nkx2-1 activates nicotinic cholinergic receptor α7 transcription (Reynolds and Hoidal, 2005).

Multiple GWASs have identified a relatively large number of single-nucleotide polymorphisms, each contributing a small risk for the disorder. A meta-analysis of 18 GWASs for schizophrenia supported involvement of the MHC region, *TCF4, POM121L2, NOTCH4, AS3MT, CNNM2*, and *NT5C2* (Aberg et al., 2013). Transcription factor 4 (TCF4) is implicated in Wnt signaling, which is critical for the maintenance the proliferation of Nkx2-1-derived GABAergic interneurons in the mouse forebrain (Gulacsi and Anderson, 2008) and for lung development (Tebar et al., 2001). Signaling through the Notch receptor may be a common regulator of neuronal differentiation within the developing forebrain (Faux et al., 2001). The Notch receptor ligand NOTCH4 is involved in lung function and remodeling as well as in protection against innate immune inflammation in the lung, where it acts on tumor necrosis factor, an upstream suppressor of NKX2-1 transcription (Boggaram, 2009; Das et al., 2011; Li C. et al., 2013). NKX2-1 in the human thyroid gland is involved in the regulation of MHC class I genes (Huang et al., 2011).

Although Nkx2-1 has been shown to interact with a number of susceptibility genes for schizophrenia, most of these studies were performed in rodents and species-related differences may occur. Many of these gene-gene interactions are also described in tissues other than the brain. However, although transcriptional targets are often tissue-specific, a significant overlap exists between transcriptional targets in different tissues. For example, Spiteri et al. (2007) reported overlaps of 47% and 37% for genes identified as forkhead box P2 (FOXP2) targets in human lung and basal ganglia, and in the lung and inferior frontal cortex, respectively. Interestingly, *FOXP2*, a candidate gene for schizophrenia, interacts with NKX2-1 in the human lung, and is co-expressed with Nkx2-1 in the posterodorsal medial amygdala nucleus in mice (Carney et al., 2010).

### NKX2-1 and the two hit hypothesis

The possible involvement of NKX2-1 in the pathogenesis and pathophysiology of schizophrenia is fully compatible with the two-hit hypothesis, underscoring the importance of environmental factors in this disease (Bayer et al., 1999; Feigenson et al., 2014). Research using transgenic mice has determined that early prenatal stress results in significant fluctuations in the expression of Nkx2-1 and its downstream targets that affect the distribution of MGE-derived interneurons in the cortex (Stevens et al., 2013). Immune activation in the fetus as a result of inflammatory stimuli emanating from the mother is proposed to represent an important environmental susceptibility factor for schizophrenia (Brown, 2011; Wischhof et al., 2015). Nkx2-1-expressing periventricular tanycytes and other cortical astrocytes in rodents respond to the inflammatory stimulus provided by lipopolysaccharide. The effects of lipopolysaccharide are at least partly mediated through regulation of the availability of triiodothyronine in the brain parenchyma, followed by changes in triiodothyronine-mediated gene expression involved in neurodevelopment and oxidative stress (Klecha et al., 2006). Prenatal administration of lipopolysaccharide is known to induce sex-dependent changes in glutamic acid decarboxylase and PV in the adult rat brain (Basta-Kaim et al., 2015). The effect of gene-environment interactions have been demonstrated in transgenic mice expressing a dominant-negative schizophrenia 1 gene; these mice display histologic and behavioral endophenotypes that are relevant to schizophrenia. When exposed to an immune challenge corresponding to viral infection during the neonatal period, the transgenic mice exhibited impaired Nkx2-1-associated behaviors, such as fear memory, social recognition, and social interaction (Ibi et al., 2010). Additive effects of the disrupted in schizophrenia 1 genotype and immune challenge were reflected in a marked decrease in the number of PV+ interneurons in the medial prefrontal cortex.

### Are dysregulated NKX2-1 associated processes specific to schizophrenia?

A central question is whether the disruption of NKX2-1associated networks and processes should be considered to be specific to the diagnostic category schizophrenia, or instead could be of broader relevance to neurodevelopmental disorders. For example, many genes identified as susceptibility genes for schizophrenia are also associated with other mental disorders (e.g., autism spectrum disorders and bipolar disorder). Overlapping symptoms are also commonly found. Psychotic symptoms can arise in bipolar disorder, and persistent social dysfunction is a prominent feature of autism. NKX2-1 expression patterns have not been explored either in schizophrenia or in any other neurodevelopmental or psychiatric disorder. However, patients with schizophrenia certainly have brain dysfunctions that involve the progeny of NKX2-1 expressing progenitor cells. Susceptibility to the somatic symptoms related to the dysfunction of physiological processes regulated by NKX2-1 are also well documented. To date, we are not aware of a similar cluster of mental and somatic dysfunction in any other mental illness, other than schizophrenia. Therefore, we would propose that aberrant NKX2-1 function could result in a wide-ranging, multi-organ disorder that is the hallmark of classic untreated “Kraepelinian” schizophrenia. However, until results of research into the role of NKX2-1 in schizophrenia, including comparisons with other highly heritable disorders are published, our proposal must be regarded as an educated hypothesis.

## Implications for research

In the past, most schizophrenia research has focused on the brain or blood. NKX2-1 is not known to be expressed in blood cells, and analyses performed on blood samples may not be feasible. However, several alternative ways of studying the role of NKX2-1 are available. Studies of genetically-engineered, experimental animals, with variable age- and tissue-specific expression of Nkx2-1, or its downstream targets, may shed light on relevant behavioral phenotypes (Fragkouli et al., 2005; Magno et al., 2011). Studies of the interactions between identified susceptibility genes and Nkx2-1 may also increase our knowledge about the mechanisms that underlie altered neurotransmission and other physiologic processes. Genes that interact with NKX2-1 also represent a new, and as yet unidentified cohort of susceptibility genes. A recent interesting GWAS identified 42 sets of single-nucleotide polymorphisms associated with a 70% or greater risk of schizophrenia (Arnedo et al., 2015). Molecular pathway analyses then indicated that processes related to neurodevelopment, immunity, neurotransmission, gene expression, and oxidative stress were all involved in the pathogenesis of schizophrenia; these are all strikingly similar to those processes involving NKX2-1. Follow-up work to assess if those single-nucleotide polymorphisms are related to NKX2-1 is now needed.

Post-mortem studies in patients with schizophrenia can be performed to study cell populations developed from NKX2-1-expressing progenitors. Analyses of the activities of NKX2-1-related gene pathways in skin biopsies or other tissues can be correlated with the symptoms and various manifestations of schizophrenia. Human pluripotent stem cells are a powerful tool with which to model brain development and disease, and it is feasible to derive cortical interneurons that express NKX2-1 in order to investigate their function under diverse experimental conditions (Maroof et al., 2013). The generation of induced pluripotent stem cells (iPSCs) from patients with schizophrenia would also enable *in vitro* electrophysiological studies of synaptic function to be conducted for iPSC-derived neurons (Bellin et al., 2012; Brennand et al., 2012). Such electrophysiological methods will enable us to study how NKX2-1 impacts synaptic function in humans, both at the single-neuron level (*in vitro*), and non-invasively, *in vivo* (in the brain). These techniques may be complemented by novel computational, in silico modeling of synaptic function. This unique approach has the potential to provide new, groundbreaking knowledge about disease mechanisms that could, potentially, inform clinical practice, and enable the personalized medicine that could improve outcomes in schizophrenia.

By linking NKX2-1 findings to electrophysiology, such studies may contribute to an improved understanding of brain oscillations in schizophrenia. Clinical studies focusing on the relationships among photic transmission, neuroendocrine anomalies, and social and emotional deficits and their association with NKX2-1, should also be performed. The hallmark mental symptoms of schizophrenia can be further investigated in the context of immune, thyroid, parathyroid, lung, skin, and gut pathology. New imaging techniques allow us to explore the impact of thyroid hormones on brain function and metabolism (Bauer et al., 2009; Pilhatsch et al., 2011), as well as visualization of distinct neural circuits (Granziera et al., 2011). New imaging techniques currently under development will also allow us to study small structures, deep in the human brain, such as the septal and amygdala subnuclei, and the hypothalamus (Prestia et al., 2015).

Although speculative, a possible implication of our emphasis on the role of the NKX2-1 transcription factor, not only for brain development, but also in relation to other physiologic defects with embryonic origins (e.g., gut, respiratory system), is that new therapies for diseases associated with those physiological defects may also be of value to patients with schizophrenia. Our hypothesis would be markedly strengthened by studies of how new drug treatments interact with NKX2-1.

## Conclusion

NKX2-1 may be involved in core, dose-sensitive, molecular pathways that are deregulated in schizophrenia. This deregulation may result from the mutation of genes that directly or indirectly interact with NKX2-1, and/or from environmental factors that influence epigenetic gene regulation. Deregulated pathways may involve both under- and over-expression, which might activate non-physiologic targets, or repress true targets. Cell type- and tissue-specific factors, together with the timing and magnitude of dysfunction, could also contribute to the phenotypic variability of schizophrenia. NKX2-1 conducts key functions at the interface of the brain, the neuroendocrine system, and the immune system, and may be an important mediator of aberrant cross-communication among these systems in schizophrenia. Knowledge of the processes that involve NKX2-1 may be of value in devising future treatment strategies for schizophrenia, including pharmacologic approaches, deep brain stimulation, and, potentially, stem cell-based treatments.

## Author contributions

EM, KJ, UM, and TN made substantial contributions to the concept and design of this work. EM drafted this manuscript. KJ, UM, and TN critically reviewed the manuscript and gave final approval of the submitted version. EM, KJ, UM, and TN are accountable for all aspects of this work and confirm that any questions related to the accuracy or integrity of any part of the work will be appropriately investigated and resolved.

### Conflict of interest statement

The authors declare that the research was conducted in the absence of any commercial or financial relationships that could be construed as a potential conflict of interest.
